# Observation of Kerr soliton microcomb locked with a phonon laser

**DOI:** 10.1126/sciadv.aeb3400

**Published:** 2026-01-02

**Authors:** Xinxin Li, Yifeng Xie, Shulin Ding, Menghua Zhang, Yaya He, Bing He, Min Xiao, Xiaoshun Jiang

**Affiliations:** ^1^ National Laboratory of Solid State Microstructures, College of Engineering and Applied Sciences, Collaborative Innovation Center of Advanced Microstructures, Nanjing University, Nanjing 210093, China.; ^2^ Multidisciplinary Center for Physics, Universidad Mayor, Camino La Pirámide 5750, Huechuraba, RM, Chile.

## Abstract

Cavity optomechanics and dissipative Kerr soliton microcomb are two research fields that followed the parallel lines of development, and the interplay of their respective elements was unseen in the past. Here, we present an observation of mutually synchronized Kerr soliton microcomb and optomechanical oscillation in the same microresonator. We generated a previously unknown type of breathing soliton to drive the mechanical mode of the microresonator into a forced oscillation at the breathing frequency. Once the breathing frequency is tuned close to the mechanical frequency of the system, the breathing soliton and the excited mechanical oscillation suddenly lock together into a synchronization. This state locks to an almost unchanged breathing frequency, and exists over a considerable frequency range of the pump laser. The locked breathing Kerr soliton can substantially lower its phase noise and improve its long-term stability, providing potential applications in various areas.

## Teaser

A mutual synchronization of Kerr soliton and optomechanical oscillation in the same microresonator.

## INTRODUCTION

With the development of high-quality factor (high-Q) optical microresonators, the nonlinear photonics in the past decade witnessed an increasingly heated study on Kerr soliton microcombs (*1*–*3*). So far, Kerr solitons microcomb generated on chip have been demonstrated in several platforms of different materials and structures (*4*–*10*) and can be even produced in a turn-key manner (*11*, *12*), promising the applications in the important aspects of modern technology (*13*, *14*). On the other hand, another important research area emerged with the coupling of optical modes and mechanical degrees of freedom of a microresonator via their mutual interaction, giving birth to the subject of cavity optomechanics (*15*, *16*). Recently, the study of cavity optomechanical systems (COMS) has led to several breakthroughs in physics and potential applications, such as ground-state cooling (*17*–*21*), quantum squeezing (*22*, *23*), gravitational wave astronomy (*24*), and ultraprecise measurements (*25*–*29*). Under suitable pump detuning and power, a COMS will stabilize in an optomechanical oscillation (OMO) (*30*–*36*). In particular, such OMO with narrow linewidth, also known as phonon lasing, offers the applications ranging from low noise signal generation (*37*) and sensing (*38*–*40*) to low–repetition rate optical frequency comb generations (*41*–*45*).

**Fig. 1.**
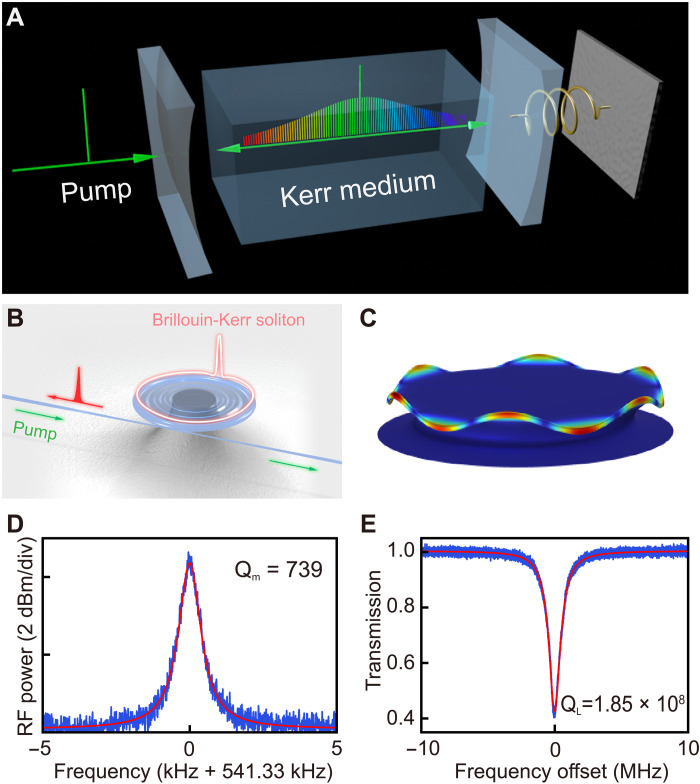
Schematic of generating Kerr soliton microcomb locked with a phonon laser. (**A**) Schematic diagram of interaction between Kerr soliton and optomechanics. (**B**) Illustration of the Kerr soliton microcomb in a COMS. (**C**) A finite-element simulation of the crown mode used in experiment. (**D**) Measured radio frequency (RF) power spectrum of the microtoroid resonator. (**E**) Optical transmission spectrum of the Brillouin mode.

A link for the two abovementioned important research areas is the Kerr soliton excited together with an OMO, as illustrated in Fig. 1A. This special type of soliton microcombs was theoretically predicted before (*46*). Although the theoretical scheme (*46*) for realizing such special optical soliton microcomb is simply based on the coupling between the multiple optical modes and the single mechanical mode, its experimental implementation has not been reported. One reason is that generating such vibrational Kerr solitons requires a large optomechanical coupling in optical microresonators, which could hinder the formation of the soliton pulses. Also, there exists a technical barrier that the generation of Kerr combs with detectable repetition rates should be with the millimeter-size microcavities of large effective mechanical mass and small optomechanical coupling coefficient, which conversely restricts the excitation of the OMO.

To overcome the abovementioned barriers, we here present and experimentally implement an approach through the generation of a previously unknown type of breathing soliton (breather) based on a Brillouin-Kerr microcomb system (*12*, *47*, *48*) and locking the breathing soliton with an OMO in the same device. The breathing frequency of the breather can be adequately adjusted by the pump laser frequency, and, once it is tuned close to the intrinsic frequency of the mechanical mode, the breather and the excited mechanical oscillation are found to be abruptly and simultaneously locked into a synchronization, and this locked state bears a similarity with the previous theoretical prediction (*46*). In contrast to previous studies on the technical synchronizations of Kerr solitons (*49*, *50*) or Kerr soliton with OMO (*51*), which are generally based on the control of the dynamics in one microresonator by the signal from the other, our observed synchronization process arises from an appropriate interaction of the intracavity-field modes with the mechanical mode in the same device.

## RESULTS

### System dynamics and setup

We use a silica microtoroid resonator (*52*, *53*), which supports Kerr nonlinearity (*54*), stimulated Brillouin scattering (SBS) (*55*), as well as cavity optomechanical effect (*30*–*32*). The Brillouin-Kerr microcomb with cavity optomechanics (Fig. 1B) can be modeled by the following equations

$$\frac{{\mathrm{da}}_{p}}{\mathrm{dt}}=\left(-i\Delta \omega_{p}-\frac{\gamma_{p}}{2}\right)a_{p}-{\mathrm{ig}}_{B}a_{b0}a+\sqrt{\kappa_{p}}s_{\text{in}}$$ (1)

$$\begin{array}{cc} \frac{{\mathrm{da}}_{\mathrm{bn}}}{\mathrm{dt}}= & \left(-i\Delta \omega_{\mathrm{bn}}-\frac{\gamma_{\mathrm{bn}}}{2}\right)a_{\mathrm{bn}}-i\delta_{0,n}g_{B}a_{p}a^{*}\\ & -{\mathrm{ig}}_{K}\sum_{k,l.m}\delta_{0,n-\left(k-l+m\right)}a_{\mathrm{bk}}a_{\mathrm{bl}}^{*}a_{\mathrm{bm}}-{\text{iGxa}}_{\mathrm{bn}} \end{array}$$ (2)

$$\frac{\mathrm{da}}{\mathrm{dt}}=\left(-i\Delta \omega_{a}-\frac{\gamma_{a}}{2}\right)a-{\mathrm{ig}}_{B}a_{p}a_{b0}^{*}$$ (3)

$$\frac{d^{2}x}{{\mathrm{dt}}^{2}}+\gamma_{m}\frac{\mathrm{dx}}{\mathrm{dt}}+\omega_{m}^{2}x=\frac{G}{m\omega_{b0}}\sum_{n}{|\;a_{\mathrm{bn}}\;|}^{2}$$ (4)

with δ_0,*n*_ being a Kronecker delta. Here, a continuous wave laser field, with its amplitude *s_in_* ($P={|\;s_{\text{in}}\;|}^{2}$ is the power) and frequency ω*_p_* detuned by Δω_p_ from the microcavity resonance frequency ω_0_, pumps the microresonator with the total decay rate $\gamma_{p}=\kappa_{p}+\gamma_{0}$ (κ_p_ is the pump-cavity coupling rate and γ_0_ the cavity intrinsic loss rate), creating the pump field mode *a*_p_. This field mode undergoes two simultaneous nonlinear dynamical processes, a four-wave mixing with the Kerr nonlinear coupling coefficient *g_K_*, and an SBS with the coupling coefficient *g*_B_. They give rise to a series of field modes *a_bn_* (*n* are integers) propagating in the reverse direction and an acoustic mode *a*, with their respective detunings $\Delta \omega_{s}=\omega_{s}-\omega_{s,0}$ and damping rates γ*_s_* for *s* = *bn* or *s* = *a*. Among them, the mode *a*_*b*0_ is the Brillouin background field. Another important feature is that these field modes can possibly drive a mechanical mode, which has an effective mass *m*, an intrinsic frequency ω*_m_*, and a damping rate γ*_m_*, into oscillation. The field force is proportional to the optomechanical coupling constant $G=\omega_{b0}/r$ (*r* is the cavity radius).

In the experiment, we use a silica microtoroid resonator with a diameter of 2.82 mm. As shown in Fig. 1E, the measured loaded Q-factor of the Brillouin mode is 1.85 × 10^8^, corresponding to an intrinsic Q-factor of 2.24 × 10^8^. Such an ultrahigh Q of the microcavity facilitates a strong coupling between the Kerr soliton and the background field (*12*). In addition, the measured mechanical Q-factor is 739 for a crown mode (see Fig. 1C) of 541.3 kHz shown in Fig. 1D.

### Generation of breathing soliton

**Fig. 2.**
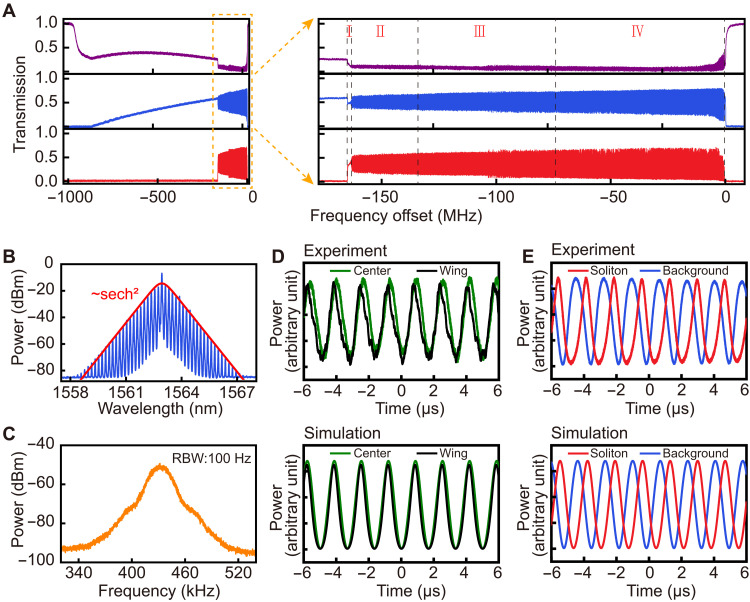
Generation of Brillouin-Kerr breathing soliton (breather). (**A**) The transmission power spectra of the pump field (the purple curve), the reverse Brillouin laser field (the blue curve), and the comb of Kerr soliton (the red curve). Here, single soliton exists in phase I, breather in phase II, phonon-laser locked Kerr soliton in phase III, and again breather in phase IV. (**B**) The optical spectrum of the generated breather (phase II). Its envelope is fitted with the red curve of the function sech^2^(*x*). (**C**) Measured RF power spectrum of the breather. RBW, resolution bandwidth. (**D**) The simultaneously evolving powers of two comb teeth at the center and at the wing, respectively. The top panel is the experimental result, and the bottom panel is from the numerical simulation with Eqs. 1 to 4. Their in-phase relation indicates that no energy exchange exists between the comb teeth. (**E**) The corresponding relation between the background light (the Brillouin laser) and the soliton. The out-of-phase oscillations indicate that there is a constant energy exchange between them.

To excite the Brillouin-Kerr soliton, we apply a pump field of 30 mW and scan its frequency from the higher to the lower. Figure 2A shows the transmission power spectra of the forward pump field (the purple curve), the reversed Brillouin laser (the background field as the blue curve), together with the Kerr comb (the red curve). It is seen that a Brillouin laser field emerges and its intensity becomes higher and higher with the lowered pump frequency. Once its power goes above the optical parametric oscillation threshold to phase I in Fig. 2A, monostable single solitons will be spontaneously generated out of the continuous background (*12*). Such shape-invariant stationary soliton, however, cannot trigger the oscillation of the mechanical mode since the magnitudes ${|\;a_{\mathrm{bn}}\;|}^{2}$ (n≠0) of its Fourier components are constant in this regime (it can be verified by numerically solving Eqs. 1 to 4) and provide no oscillatory driving force on the right-hand side of Eq. 4.

After the pump frequency is lowered further, the Kerr soliton will enter a breathing state (phase II in Fig. 2A), in which the output field mode powers of the single soliton microcomb become oscillatory (the Fourier component magnitudes ${|\;a_{\mathrm{bn}}\;|}^{2}$ become periodically oscillating). The breather is firstly characterized by the measured optical spectrum in Fig. 2B. In contrast to the previously reported breathers with a triangle spectrum envelope (*56*, *57*), the comb teeth of our breather are always enveloped by the function sech^2^(*x*); see the fitted curve in Fig. 2B and more information in the Supplementary Materials. Here, the formation of this kind of breather are triggered by the energy exchange between the soliton and the background field. We verify this fact by both experimental observations and numerical simulation. From Fig. 2D, one sees that the power evolutions of two comb teeth, which are respectively at the center and at the wing of the microcomb spectrum, oscillate in the same phase, implying that the breather does not arise from the intrinsic dynamical instability in Kerr microresonators (*56*). Besides the oscillation of the generated Kerr soliton, the power of the background light simultaneously oscillates as shown in Fig. 2E, but its out-of-phase oscillation with respect to the soliton power indicates a periodic energy exchange between them.

In the state of breather, the oscillation of the field modes is characterized by the breathing frequency, as it is deduced from the measured radio frequency spectrum in Fig. 2C. It is known from a numerical simulation based on Eqs. 1 to 4 that the summation of ${|\;a_{\mathrm{bn}}\;|}^{2}$ on the right-hand side of Eq. 4 is dominated by the components oscillating at the breathing frequency ω*_b_* so that Eq. 4 can be written in the following approximate form as a forced mechanical oscillation

$$\frac{d^{2}x}{{\mathrm{dt}}^{2}}+\gamma_{m}\frac{\mathrm{dx}}{\mathrm{dt}}+\omega_{m}^{2}x=\frac{F_{B}}{m}\text{cos}\left(\omega_{b}t+\phi_{B}\right)+\frac{F_{S}}{m}\text{cos}\left(\omega_{b}t+\phi_{S}\right)$$ (5)

Here, the oscillatory Brillouin laser and breathing soliton field, with their force amplitudes *F_B_* and *F_S_*, as well as with their phase ϕ*_B_* and ϕ*_S_*, respectively, drive the mechanical mode. The mechanical mode will respond to these driving forces and oscillate at the breathing frequency too. Moreover, the breathing frequency can be adjusted by the pump detuning (*57*, *58*), and this property is highly useful to our experiment.

### Synchronization of breather and OMO

**Fig. 3.**
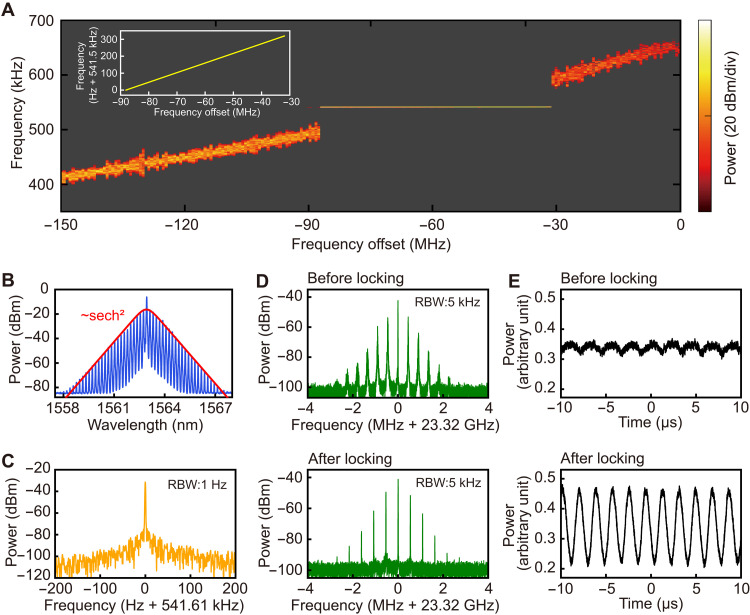
Creation and characteristics of the phonon-laser–locked breather. (**A**) The measured real-time variation of the breathing frequency along with the pump frequency scan. The initially increasing breathing frequency is seen to be locked to the mechanical frequency of the microresonator within the observed range of pump detuning. Inside the range of frequency locking, the frequency of the excited OMO only changes by a few hundred hertz due to the optical spring effect, as seen from the inset. (**B**) Optical spectrum of the phonon-laser–locked breather with its envelope fitted to the form of the function sech^2^(*x*) (the red curve). (**C**) Measured RF power spectrum of the locked breather. (**D**) Repetition rate beat note spectra of the locked and the unlocked breathers. (**E**) The comparison of the probe transmission powers for the state of locked and unlocked breathers.

By means of carefully tuning the breathing frequency of the breather via tuning the pump frequency, we experimentally observe an interesting phenomenon: The breathing frequency will be suddenly locked when it gets close to the intrinsic mechanical frequency of the microtoroid resonator. Figure 3A presents the evolution of the breathing frequency with the decreased pump frequency. Initially, the breathing frequency increases in an approximately linear way. However, as the breathing frequency reaches a point close to the intrinsic mechanical frequency, its value will be suddenly locked to an almost fixed one in phase III of Fig. 2A, and the system will keep this locked state as long as the pump detuning is within the observed frequency window. The breathing frequency will not restore the linearly increasing tendency until the system goes beyond the locking range and into phase IV (a breather state with the breathing frequency larger than the mechanical frequency of the system) in Fig. 2A. Within the range of frequency locking, the mechanical oscillation frequency only changes by a few hundred hertz due to the existing optical spring effect; see the details in the inset of Fig. 3A. In this locked state, the measured optical spectrum of the breathing soliton microcomb still preserves an envelope in the form of the function sech^2^(*x*) (see Fig. 3B).

To further understand the locked state of the breathing soliton and the phonon laser, we have performed the numerical simulations based on Eqs. 1 to 4 and compared the simulations with the measured results. Before the locking of the breathing frequency, the background light (the Brillouin laser) does a positive work in each mechanical oscillation period, while the breather does a negative work at the same time since their oscillations are relatively out-of-phase to each other (see the Supplementary Materials). In other words, the forced mechanical oscillation in phase II of Fig. 2A is mainly sustained by the background field. However, the breathing frequency locking suddenly reverses the roles of the background light and soliton, i.e., the phases ϕ_B_ and ϕ_S_ of their corresponding forces in Eq. 5 will be suddenly changed; see fig. S3 in the Supplementary Materials. Now the soliton contributes to the positive work instead (see the Supplementary Materials), and it will significantly alter the system dynamics. Also, in both locked and unlocked state, the net works of the background field and the breathing soliton are positive to overcome the mechanical loss, but the difference is that the mechanical mode is in a forced oscillation before the locking and it enters a phonon lasing state after the locking. A clearer difference of the locked breathing soliton from the unlocked breather is seen from Fig. 3C: The linewidths of the breathing frequency in the locked state have been tremendously reduced to the order of 1 Hz, corresponding to the ultranarrow linewidth of the OMO as a phonon laser, which is much narrower than that of the unlocked breather (Fig. 2C). The generation of the breather results in additional sidebands around the repetition rate of the microcomb. As shown in Fig. 3D, the sidebands of the locked breather exhibit much narrower linewidths as compared with those of the unlocked one. In the locked state, the system oscillates at its mechanical frequency rather than a variable breathing frequency of the soliton. Moreover, the induced mechanical oscillation will increase its amplitude by one order due to the resonance effect of the driving force; see Supplementary Materials. To confirm this result, we experimentally apply a weak probe light at the wavelength of 1587.5 nm (far from the soliton spectrum) into the microcavity and measure the dynamic response of the mechanical oscillation (*59*). As shown in Fig. 3E, the measured modulation depth of the probe light for the locked state (phase III) is much larger than that of the unlocked state, which indicates that the oscillation amplitude of the phonon laser is around one order larger than that of the forced mechanical oscillation before locking.

The phenomena of synchronization into locked dynamical patterns widely exist in the systems modeled in terms of nonlinearly coupled oscillators (*60*). More relevant examples have been seen from the processes involving either Kerr solitons or OMOs (*49*–*51*, *61*–*63*). Our observed phenomenon of the mutually locked breathing soliton and narrow-linewidth OMO must occur under two prerequisites. First, a breather of sufficient oscillation intensity should be created through an adequate energy exchange with the background light so that an OMO can be excited in the regime of frequency locking (see the Supplementary Materials). Second, also through changing the pump detuning, one must be able to tune the breathing frequency across a wide range covering the intrinsic mechanical frequency of the microresonator. Only under these proper conditions will the multiple field modes *a_bn_* (n≠0) of the breathing Kerr soliton be locked together with the excited mechanical mode into a collective state—the narrow-linewidth phonon lasing and the Kerr soliton breathing at the locked mechanical frequency.

### Properties of locked breather

**Fig. 4.**
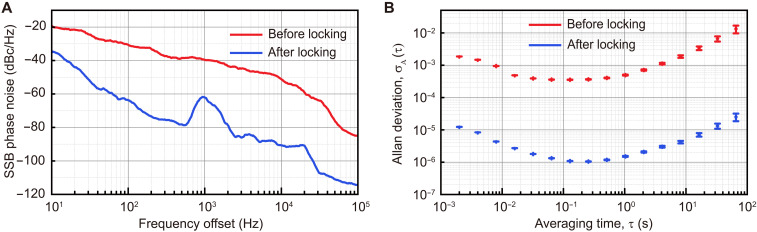
Comparison of the phase noise and stability of the unlocked and locked breathers. (**A**) The single-sideband phase noise of the breathing frequency for the unlocked (the red curve) and locked (the blue curve) breather, respectively. (**B**) The Allan deviations of the breathing frequency for the unlocked (the red points) and locked (the blue points) breather, respectively.

More features of the locked breather are demonstrated in Fig. 4. Here, we present the phase noise of the breathing frequency before and after locking. We find that the phase noise of the breather will be significantly suppressed once the system enters the regime of frequency locking, where the breather linewidth is significantly reduced to the linewidth of OMO. More exactly, as seen from the measurement in Fig. 4A, the locked breather has a much lower phase noise −62 dBc/Hz at the frequency offset 1 kHz, in contrast to the phase noise −39 dBc/Hz of the unlocked breather at the same frequency offset. At another frequency offset of 10 kHz, the phase noise becomes −51 dBc/Hz for the unlocked breather and −90 dBc/Hz for the locked breather, respectively. Note that the peak at ~1 kHz offset frequency of the phase noise spectrum for the locked breather is attributed to the frequency noise of the pump laser. The phase noise of the breather can be further reduced by increasing the mechanical Q-factor of the system. To quantity their long-term stability, we measure the Allan deviations for the locked and unlocked breather, respectively, as shown in Fig. 4B. It is found that the Allan deviation of the locked breather is improved by two orders as compared with that of the unlocked breather.

## DISCUSSION

We have presented an observation of the mutually locked Kerr soliton and OMO in a microresonator. The locked state is realized through a sophisticated scheme to generate a previously unknown type of breather that was not observed before. The formation of this type of breather is associated with an energy exchange between the background field and the soliton, and its more complete generation mechanism will be explored further. The breathing frequency of the breather can be tuned over a wide range via the pump detuning. Once the breathing frequency is tuned close to the mechanical frequency of the microresonator, the breather will be locked together with the mechanical oscillation simultaneously excited by the intracavity field. Their mutual locking gives rise to the locked breathing soliton with improved stability. In addition to the potential applications of the locked breather such as soliton dual microcomb with increased resolution (*64*), the experiment marks a stage that two important research fields, cavity optomechanics and Kerr soliton microcomb, can be meaningfully joined to explore new physics. The further progress may develop subharmonic entrainment in breathing soliton microcomb (*65*), pulsed optomechanics (*66*) and include mechanical solitons (*67*).

## MATERIALS AND METHODS

### Silica microtoroid resonator fabrication

We fabricate the silica microtoroid resonator based on the previously developed process (*52*, *53*). We first fabricate a silica microdisk (diameter = 3 mm) by the standard photolithography and buffered HF solution etching upon a silicon wafer with a 12-μm layer of SiO_2_. The microdisk is then undercut by using XeF_2_ dry etching to create a circular silicon pedestal. The edge of the disk is reflowed to a toroid shape by a CO_2_ laser with the power of 48 W in a nitrogen ambience. We circulate the laser around the periphery of the disk by using a three-dimensional linear translation stage due to the limited beam waist of the CO_2_ laser. To further improve the optical quality factor of the microresonator, the microtoroid has undergone a 36-hour annealing at 1000°C in an oxygen ambience.

## Supplementary Materials

### This PDF file includes:

Supplementary Text Figs. S1 to S4 References
