# Supplementary material for: Observation of Kerr soliton microcomb locked with a phonon laser
Source: Sci Adv. 2026 Jan 2;12(1):eaeb3400. doi: 10.1126/sciadv.aeb3400 (PMC12758527; doi:10.1126/sciadv.aeb3400)
Supplement: Supplementary file 1 — Supplementary Text Figs. S1 to S4 References [file sciadv.aeb3400_sm.pdf]

Science Advances
AAAS

Supplementary Materials for

Observation of Kerr soliton microcomb locked with a phonon laser

Xinxin Li *et al.*

Corresponding author: Bing He, bing.he@umayor.cl; Xiaoshun Jiang, jxs@nju.edu.cn

Sci. Adv. **12**, eaeb3400 (2026)
DOI: 10.1126/sciadv.aeb3400

This PDF file includes:

Supplementary Text
Figs. S1 to S4
References

Supplementary Text

Experimental Setup

The experimental setup for generation of Kerr soliton microcomb locked with a phonon laser is shown in Fig. S1. The pump laser at 1562 nm is amplified by an erbium-doped fiber amplifier and then coupled into the silica microtoroid through a fiber taper. A variable optical attenuator (VOA) and a fiber polarization controller (FPC) are used to conveniently adjust the power and polarization of the pump laser. The power meter (PM) is used to monitor the pump power coupled into the microcavity. The forward transmission spectrum signal is measured by photodetector PD1, and the backward signal is measured by photodetector PD2, and the comb signal is measured by photodetector PD3, respectively. The signals from these three photodetectors are connected to an oscilloscope (OSC) for monitoring, allowing for an initial assessment of the soliton microcomb state. An optical spectrum analyzer (OSA) is used to observe the soliton spectrum, and an electrical spectrum analyzer (ESA) is used to measure the breathing frequency.

Determination of the System Parameters

The simulations of the dynamical processes are based on Eq. (1)-Eq. (4) in the main text, which were developed from our previous works(12, 48). We numerically solve Eqs. (1)–(4) by using the fourth-order Runge-Kutta method and compare our results with the experimental findings. In the simulations we consider 256 modes and employ a Fourier transform-based computationally efficient approach (68) to deal with the four-wave mixing term $g_K \sum_{k,l.m} \delta_{0,n-(k-l+m)} a_{bk} a^*_{bl} a_{bm}$ in Eq. (2). Different from a preset soliton configuration in a previous theoretical study (46), the intracavity field is set to be zero at $t = 0$. Moreover, to trigger the Brillouin lasing and Kerr comb generation, we introduce the quantum vacuum fluctuations modeled as stochastic Langevin noise.

We first determine the parameters for the three essential elements: (1) the mechanical oscillation mode that can be excited by the intracavity field; (2) the four-wave mixing (FWM) for the generation of Kerr comb; (3) the stimulated Brillouin scattering (SBS) for the generation of the CW background Brillouin laser. The optomechanical coupling coefficient is calculated as $G = \omega_{b0}/r = 2\pi \times 1.3 \times 10^{17}$ Hz/m, where $r = 1.47$ mm is the radius of the fabricated cavity. The intrinsic mechanical frequency $\omega_m = 2\pi \times 541$ kHz and $\gamma_m = 2\pi \times 732$ Hz are measured as in Fig. 3 and Fig. 1 of

the main text, respectively. Considering the microtoroid's geometry and finite-element simulation result in COMSOL, we use an effective mode area $A_{eff} = 155\ \mu m^2$ and $r = 1.47$ mm to find the Brillouin mode's effective volume as $V_{eff} = 1.4310^{-12} m^3$ and the mechanical oscillator's effective mass as $m = 871\ \mu$g. By using silica material parameters and the reference (48), we determined the Kerr coefficient as $g_K = 2.64 \times 10^{15}$ Hz/J. Moreover, with a pump-Brillouin mode overlap coefficient of 0.19, the Brillouin gain coefficient was estimated as $g_B = 1.74 \times 10^{12}$ Hz/J$^{1/2}$.

For the resonant Brillouin mode and Kerr comb modes, there is the relation $\omega_{bn,0} = \omega_{b0,0} + D_1 n + \frac{1}{2} D_2 n^2$, where $D_1/2\pi$ and $D_2/2\pi$ denote the free spectral range and second-order dispersion of the microresonator, respectively. The resonance frequency mismatch among the pump, Brillouin, and acoustic mode is set to be $\Delta\omega_0 = \omega_0 - \omega_{b0,0} - \omega_{a,0} = -2\pi \times 25$ MHz. Under our experimental conditions, we have $D_2 = 2\pi \times 180$ kHz and the damping rates $\gamma_0 = 2\pi \times 2.03$ MHz and $\gamma_{bn} = 2\pi \times 0.85$ MHz for the pumping mode and the comb modes, respectively. The coupling loss rates for the pump mode in the experiment is $\kappa_p = 2\pi \times 11.41$ MHz. In addition, the acoustic mode loss rate is determined from the material property to be $\gamma_a = 2\pi \times 30$ MHz (12).

More Information about the Brillouin-Kerr Breathing Soliton

In the previous research (56, 57), the breathing soliton arises from the intrinsic dynamical instability in Kerr microresonators, which is related to Fermi-Pasta-Ulam recurrence. The phenomenon that the power change of the comb lines around the center is nearly out of phase with the comb lines in the wings of the comb is a signature of FPU recurrence (56). As the result of averaging the oscillating comb by the optical spectrum analyser, the spectrum will feature a triangular envelope. In our case, however, the soliton has all comb lines simultaneously exchanging the energy with the background field, which is an extra dynamical element in our process. To better clarify this point, we performed numerical simulations showing the transient optical spectra of the breather over half of its breathing period, as presented in Fig. S2. One can see that the soliton always has a spectrum with a $\mathrm{sech}^2(x)$ envelope, so the average spectrum over the period maintains a $\mathrm{sech}^2(x)$ envelope.

Comparison between the Locked and Unlocked State

To better understand what differences have been realized for the locked Kerr soliton with a phonon laser, we performed the numerical simulations to compare the corresponding electrical spectra,

the displacements of the mechanical oscillation, as well as the cumulative works performed by each intracavity component in Fig. S3. As illustrated in the first and second rows of Fig. S3, when the system is in a state before being locked to a synchronization, the mechanical oscillator simply undergoes a forced oscillations driven by the periodic optical field forces. Its displacement amplitude is approximately 0.2 pm, and its oscillation frequency matches the one of the external driving forces which is around 478 kHz. In contrast, after the intracavity field and the forced mechanical oscillation are locked together as in Fig. 3 of the main text, the mechanical oscillation frequency is seen to approach the mechanical frequency 541.1 kHz of the system. Since the field components providing the driving forces are also modulated to the mechanical frequency, the mechanical displacement amplitude becomes nearly 30 times larger, reaching approximately 6 pm. Here the 100 Hz difference from the exact mechanical frequency (541 kHz) is due to an optical spring effect.

It is remarkable that although a larger optomechanical force would result in a larger mechanical oscillation amplitude, it will not cause such significant increase of the mechanical amplitude after locking. According to the simulation, the optomechanical force in phase III (the locked state) increases by 13 % than that in phase II (an unlocked state), and the corresponding optomechanical force in phase IV (another unlocked state) is even 11 % larger than that in phase III (the locked state). However, the mechanical oscillation amplitude in the locked phase III is much larger than those in both phase II and phase IV. Therefore, such a significant increase in amplitude is not due to the increase of optomechanical force. The microcavity is in a forced oscillation when it is driven periodically by the breathing soliton. In the locked state, the breathing frequency reaches the eigen frequency of the mechanical mode, so the mechanical oscillation amplitude increases significantly due to the resonance effect.

In addition, we compute the cumulative work done by each field force component over two oscillation periods. Based on Eqs. (1)-(4) in the main text, we find the light-field force contributions from the Brillouin mode and the comb modes. Then, by integrating the product of the instantaneous force and the velocity over time, the work done by each force component can be determined, as shown in the third row of Fig. S3. There, the vertical dashed lines indicate the boundaries of one oscillation period, while the horizontal dashed lines represent the zero work. These results reveal the significant differences in the energy transfer before and after the mutual locking of the

intracavity field and mechanical oscillation. In an unlocked state, the Brillouin mode plays the role of the primary driving force to do positive work per oscillation cycle, while the comb modes are doing the negative work. However, after the system is locked, their roles will be reversed: the comb modes contribute to a positive work, while the Brillouin mode does a negative work. In terms of Eq. (5) of the main text, it is to have a sudden change of the phases ϕ_B and ϕ_S. This simulation further confirms that the mechanical oscillation at the mechanical frequency, i.e. the phonon lasing of narrow linewidth, is indeed excited by the mutually locked breathing soliton. Moreover, in both situations, the absolute values of the work done by the frictional force (the green curve) keeps equal to the total net work by the intracavity field (the orange curve) at the termination of each oscillation period, reflecting the energy conservation in the steady oscillations.

To experimentally verify the phase relationship between the Brillouin laser and the comb with the displacement of the oscillator before and after the locking, we use a weak probe light far from the soliton spectrum. Figure S4 shows the phase relationship of the Brillouin laser, the comb and probe laser before and after the mutual locking of the breathing soliton and optomechanical oscillation. One sees that before the locking, the Brillouin laser is in-phase with the probe laser, while the comb is out-of-phase with the probe laser. However, after the locking, the Brillouin laser becomes out-of-phase with the probe laser, while the comb changes to in-phase with the probe laser. These observational results show a good consistency with the previous simulations.

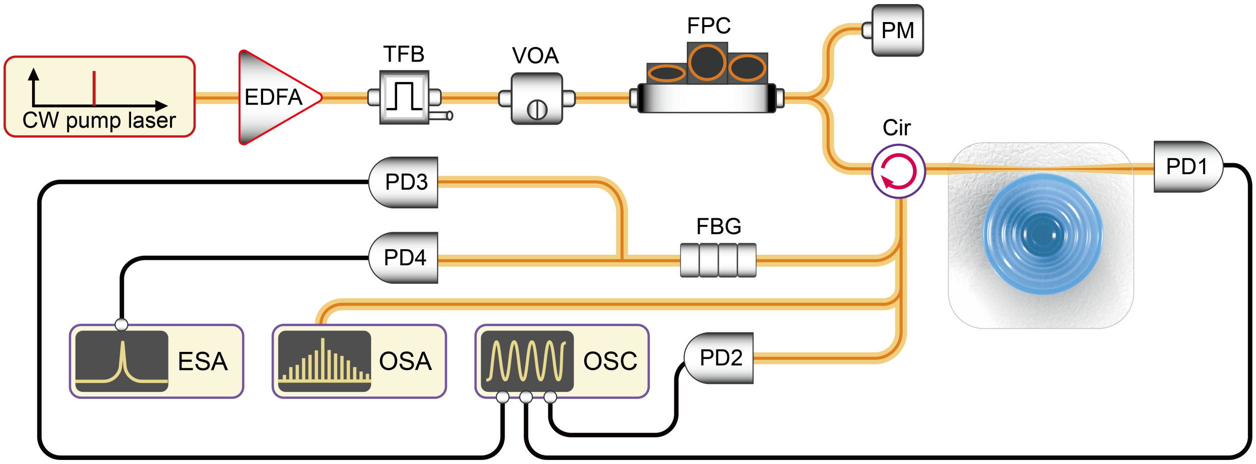

Figure S1: **Experimental setup for generation of Kerr soliton microcomb locked with a phonon laser.** EDFA, erbium-doped fiber amplifier; TBF, tunable bandpass filter; VOA, variable optical attenuator; FPC, fiber polarization controller; PM, power meter; Cir, circulator; PD, photodiode; FBG, fiber Bragg grating; OSC, oscilloscope; OSA, optical spectrum analyzer; ESA, electric spectrum analyzer.

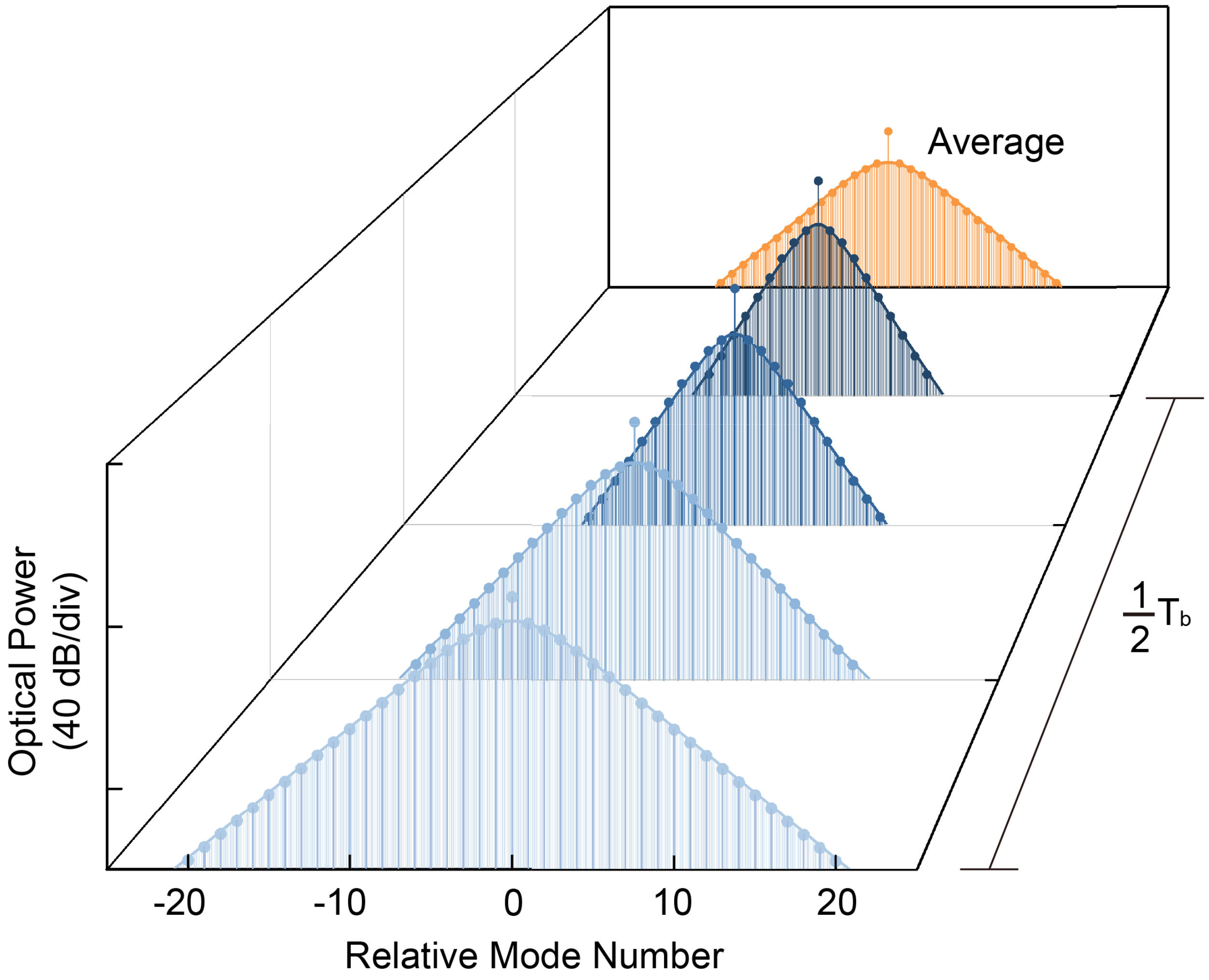

Figure S2: **Simulations of the breathing soliton spectrum at different moments.**

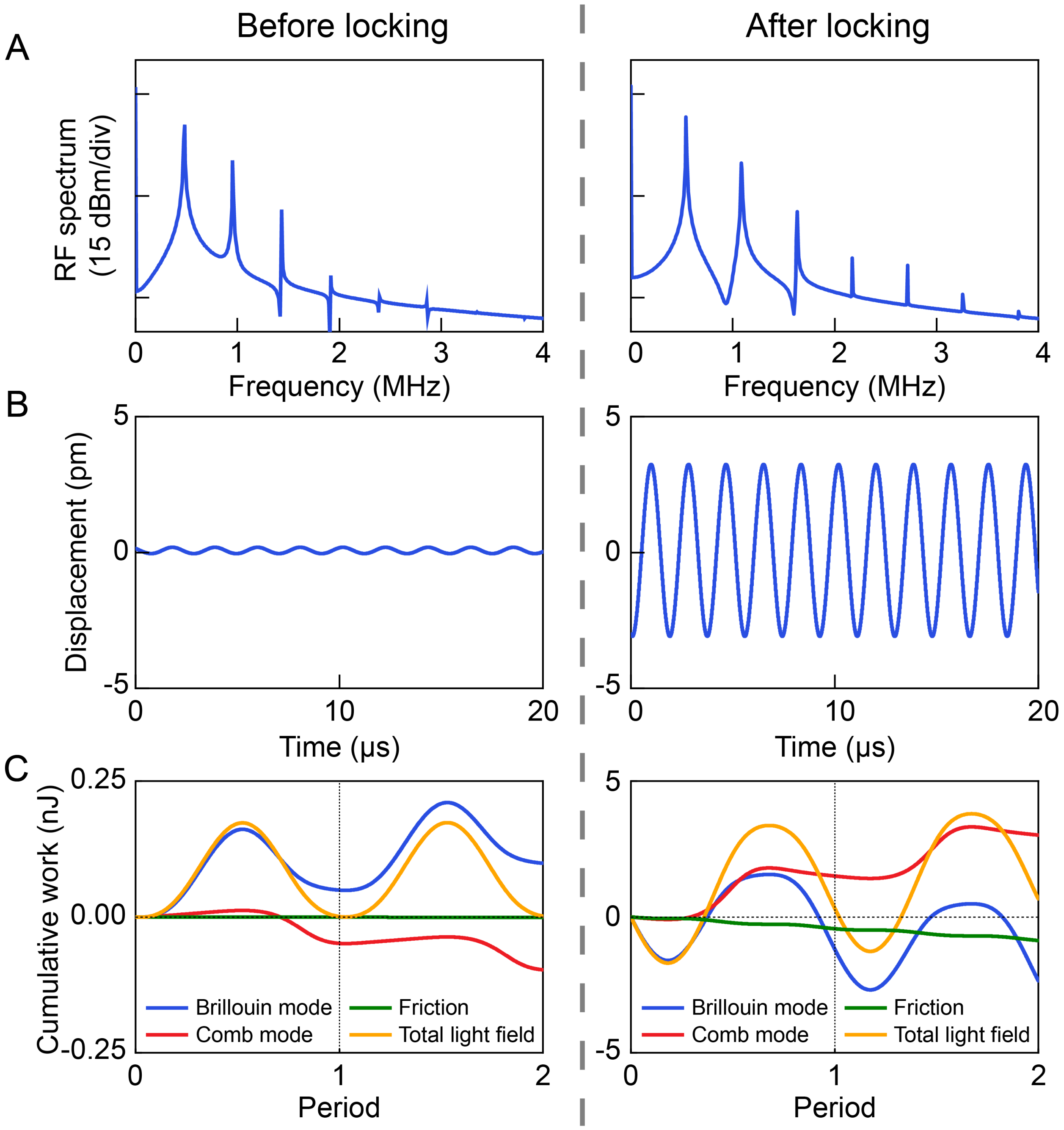

Figure S3: **Comparison between the states before and after locking.** **(A)** Radio-frequency spectrum. **(B)** Mechanical oscillation displacement. **(C)** Cumulative work done by the relevant forces within two oscillation periods. The blue curve: the Brillouin mode; the red curve: the comb modes; the green curve: the mechanical friction force; the orange curve: the total intracavity field. At the end of each oscillation period, the absolute value of the orange curve is the same as that of the green curve, but their quantities have the opposite sign.

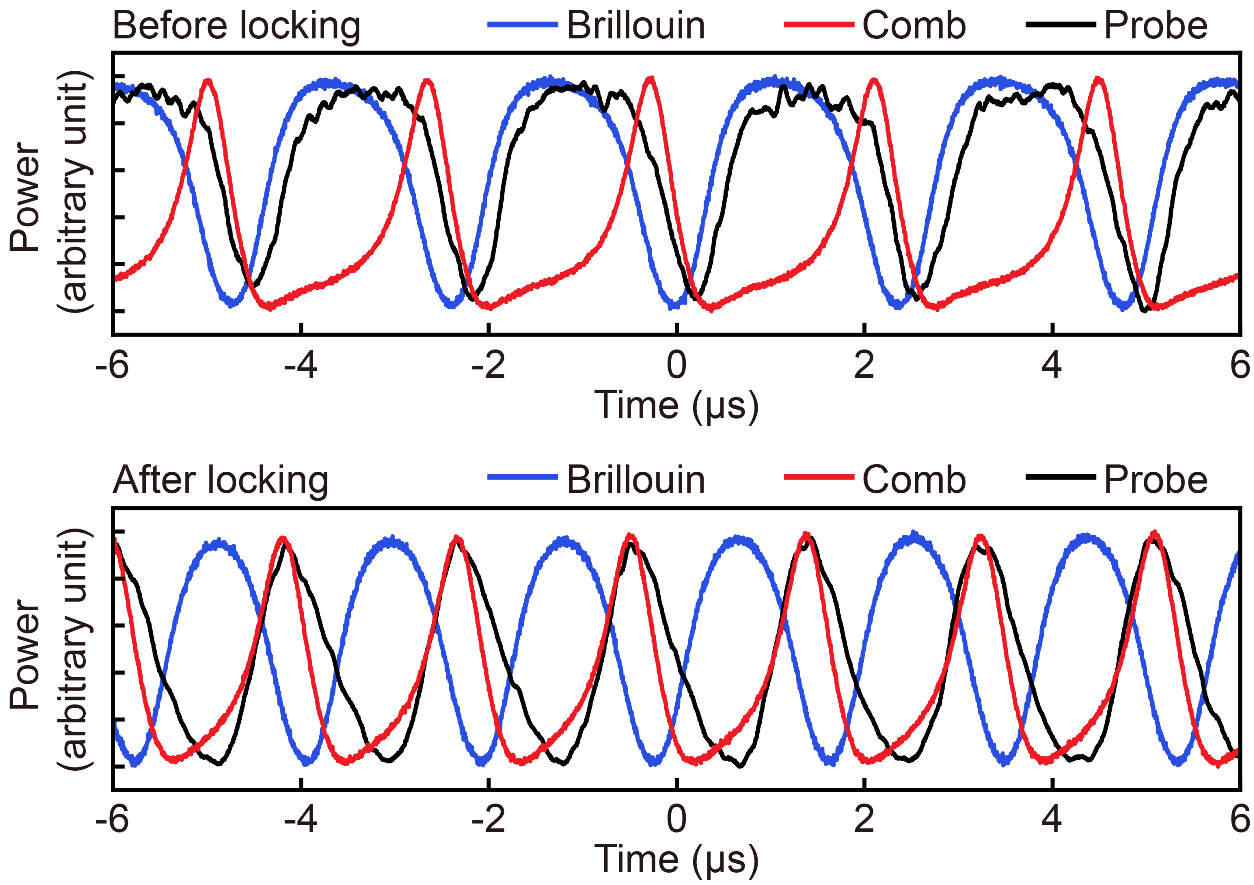

Figure S4: **Phase of the Brillouin laser (the blue curve), the comb (the red curve) and the probe laser (the black curve) before and after locking.**

REFERENCES

1. T. J. Kippenberg, A. L. Gaeta, M. Lipson, M. L. Gorodetsky, Dissipative Kerr solitons in optical microresonators. *Science* **361**, eaan8083 (2018).

2. A. Pasquazi, M. Peccianti, L. Razzari, D. J. Moss, S. Coen, M. Erkintalo, Y. K. Chembo, T. Hansson, S. Wabnitz, P. Del'Haye, X. Xue, A. M. Weiner, R. Morandotti, Micro-combs: A novel generation of optical sources. *Phys. Rep.* **729**, 1–81 (2018).

3. S. A. Diddams, K. Vahala, T. Udem, Optical frequency combs: Coherently uniting the electromagnetic spectrum. *Science* **369**, eaay3676 (2020).

4. X. Yi, Q.-F. Yang, K. Y. Yang, M.-G. Suh, K. Vahala, Soliton frequency comb at microwave rates in a high-Q silica microresonator. *Optica* **2**, 1078–1085 (2015).

5. V. Brasch, M. Geiselmann, T. Herr, G. Lihachev, M. H. Pfeiffer, M. L. Gorodetsky, T. J. Kippenberg, Photonic chip-based optical frequency comb using soliton Cherenkov radiation. *Science* **351**, 357–360 (2016).

6. Z. Gong, A. Bruch, M. Shen, X. Guo, H. Jung, L. Fan, X. Liu, L. Zhang, J. Wang, J. Li, J. Yan, H. X. Tang, High-fidelity cavity soliton generation in crystalline AlN micro-ring resonators. *Opt. Lett.* **43**, 4366–4369 (2018).

7. Y. He, Q.-F. Yang, J. Ling, R. Luo, H. Liang, M. Li, B. Shen, H. Wang, K. Vahala, Q. Lin, Self-starting bi-chromatic $LiNbO_3$ soliton microcomb. *Optica* **6**, 1138–1144 (2019).

8. G. Moille, L. Chang, W. Xie, A. Rao, X. Lu, M. Davanço, J. E. Bowers, K. Srinivasan, Dissipative Kerr solitons in a III-V microresonator. *Laser Photonics Rev.* **14**, 2000022 (2020).

9. D. Xia, Z. Yang, P. Zeng, B. Zhang, J. Wu, Z. Wang, J. Zhao, J. Huang, L. Luo, D. Liu, S. Yang, H. Guo, Z. Li, Integrated chalcogenide photonics for microresonator soliton combs. *Laser Photonics Rev.* **17**, 2200219 (2023).

10. Z. Yuan, M. Gao, Y. Yu, H. Wang, W. Jin, Q.-X. Ji, A. Feshali, M. Paniccia, J. Bowers, K. Vahala, Soliton pulse pairs at multiple colours in normal dispersion microresonators. *Nat. Photonics* **17**, 977–983 (2023).

11. B. Shen, L. Chang, J. Liu, H. Wang, Q.-F. Yang, C. Xiang, R. N. Wang, J. He, T. Liu, W. Xie, J. Guo, D. Kinghorn, L. Wu, Q.-X. Ji, T. J. Kippenberg, K. Vahala, J. E. Bowers, Integrated turnkey soliton microcombs. *Nature* **582**, 365–369 (2020).

12. M. Zhang, S. Ding, X. Li, K. Pu, S. Lei, M. Xiao, X. Jiang, Strong interactions between solitons and background light in Brillouin-Kerr microcombs. *Nat. Commun.* **15**, 1661 (2024).

13. Y. Sun, J. Wu, M. Tan, X. Xu, Y. Li, R. Morandotti, A. Mitchell, D. J. Moss, Applications of optical microcombs. *Adv. Opt. Photonics* **15**, 86–175 (2023).

14. H. Shu, B. Shen, H. Chang, J. Han, J. Xiao, X. Wang, Microcomb technology: From principles to applications. *Photon. Insights* **3**, R09 (2024).

15. G. J. Milburn, M. J. Woolley, An introduction to quantum optomechanics. *Acta Phys. Slovaca* **61**, 483–601 (2011).

16. M. Aspelmeyer, T. J. Kippenberg, F. Marquardt, Cavity optomechanics. *Rev. Mod. Phys.* **86**, 1391–1452 (2014).

17. I. Wilson-Rae, N. Nooshi, W. Zwerger, T. J. Kippenberg, Theory of ground state cooling of a mechanical oscillator using dynamical backaction. *Phys. Rev. Lett.* **99**, 093901 (2007).

18. F. Marquardt, J. P. Chen, A. A. Clerk, S. M. Girvin, Quantum theory of cavity-assisted sideband cooling of mechanical motion. *Phys. Rev. Lett.* **99**, 093902 (2007).

19. J. D. Teufel, T. Donner, D. Li, J. W. Harlow, M. S. Allman, K. Cicak, A. J. Sirois, J. D. Whittaker, K. W. Lehnert, R. W. Simmonds, Sideband cooling of micromechanical motion to the quantum ground state. *Nature* **475**, 359–363 (2011).

20. J. Chan, T. P. M. Alegre, A. H. Safavi-Naeini, J. T. Hill, A. Krause, S. Gröblacher, M. Aspelmeyer, O. Painter, Laser cooling of a nanomechanical oscillator into its quantum ground state. *Nature* **478**, 89–92 (2011).

21. B. He, L. Yang, Q. Lin, M. Xiao, Radiation pressure cooling as a quantum dynamical process. *Phys. Rev. Lett.* **118**, 233604 (2017).

22. D. W. C. Brooks, T. Botter, S. Schreppler, T. P. Purdy, N. Brahms, D. M. Stamper-Kurn, Non-classical light generated by quantum-noise-driven cavity optomechanics. *Nature* **488**, 476–480 (2012).

23. A. H. Safavi-Naeini, S. Gröblacher, J. T. Hill, J. Chan, M. Aspelmeyer, O. Painter, Squeezed light from a silicon micromechanical resonator. *Nature* **500**, 185–189 (2013).

24. H. Miao, Y. Ma, C. Zhao, Y. Chen, Enhancing the bandwidth of gravitational-wave detectors with unstable optomechanical filters. *Phys. Rev. Lett.* **115**, 211104 (2015).

25. J. D. Teufel, T. Donner, M. A. Castellanos-Beltran, J. W. Harlow, K. W. Lehnert, Nanomechanical motion measured with an imprecision below that at the standard quantum limit. *Nat. Nanotechnol.* **4**, 820–823 (2009).

26. E. Gavartin, P. Verlot, T. J. Kippenberg, A hybrid on-chip optomechanical transducer for ultrasensitive force measurements. *Nat. Nanotechnol.* **7**, 509–514 (2012).

27. A. G. Krause, M. Winger, T. D. Blasius, Q. Lin, O. Painter, A high-resolution microchip optomechanical accelerometer. *Nat. Photonics* **6**, 768–772 (2012).

28. S. Forstner, S. Prams, J. Knittel, E. D. van Ooijen, J. D. Swaim, G. I. Harris, A. Szorkovszky, W. P. Bowen, H. Rubinsztein-Dunlop, Cavity optomechanical magnetometer. *Phys. Rev. Lett.* **108**, 120801 (2012).

29. M. Sansa, M. Defoort, A. Brenac, M. Hermouet, L. Banniard, A. Fafin, M. Gely, C. Masselon, I. Favero, G. Jourdan, S. Hentz, Optomechanical mass spectrometry. *Nat. Commun.* **11**, 3781 (2020).

30. H. Rokhsari, T. J. Kippenberg, T. Carmon, K. J. Vahala, Radiation-pressure-driven micro-mechanical oscillator. *Opt. Express* **13**, 5293–5301 (2005).

31. T. Carmon, H. Rokhsari, L. Yang, T. J. Kippenberg, K. J. Vahala, Temporal behavior of radiation-pressure-induced vibrations of an optical microcavity phonon mode. *Phys. Rev. Lett.* **94**, 223902 (2005).

32. T. J. Kippenberg, H. Rokhsari, T. Carmon, A. Scherer, K. J. Vahala, Analysis of radiation-pressure induced mechanical oscillation of an optical microcavity. *Phys. Rev. Lett.* **95**, 033901 (2005).

33. J. B. Khurgin, M. W. Pruessner, T. H. Stievater, W. S. Rabinovich, Optically pumped coherent mechanical oscillators: The laser rate equation theory and experimental verification. *New J. Phys.* **14**, 105022 (2012).

34. S. Christou, V. Kovanis, A. E. Giannakopoulos, Y. Kominis, Parametric control of self-sustained and self-modulated optomechanical oscillations. *Phys. Rev. A* **103**, 053513 (2021).

35. J. Zhang, M. Orszag, M. Xiao, X. Jiang, Q. Lin, B. He, Highly correlated optomechanical oscillations manifested by an anomalous stabilization. *Phys. Rev. Lett.* **133**, 103602 (2024).

36. S. Ding, B. He, Y. Wu, Y. Hu, H. Wang, W. Wan, M. Xiao, X. Jiang, Bloch-band structure of cavity optomechanical oscillations. *Phys. Rev. Res.* **7**, L012059 (2025).

37. H. Rokhsari, M. Hossein-Zadeh, A. Hajimiri, K. Vahala, Brownian noise in radiation-pressure-driven micromechanical oscillators. *Appl. Phys. Lett.* **89**, 261109 (2006).

38. F. Liu, S. Alaie, Z. C. Leseman, M. Hossein-Zadeh, Sub-pg mass sensing and measurement with an optomechanical oscillator. *Opt. Express* **21**, 19555–19567 (2013).

39. W. Yu, W. C. Jiang, Q. Lin, T. Lu, Cavity optomechanical spring sensing of single molecules. *Nat. Commun.* **7**, 12311 (2016).

40. B. Guha, P. E. Allain, A. Lemaître, G. Leo, I. Favero, Force sensing with an optomechanical self-oscillator. *Phys. Rev. Appl.* **14**, 024079 (2020).

41. M.-A. Miri, G. D'Aguanno, A. Alù, Optomechanical frequency combs. *New J. Phys.* **20**, 043013 (2018).

42. Y. Hu, S. Ding, Y. Qin, J. Gu, W. Wan, M. Xiao, X. Jiang, Generation of optical frequency comb via giant optomechanical oscillation. *Phys. Rev. Lett.* **127**, 134301 (2021).

43. Q. Lin, Y. Wu, G. Li, B. He, Nonlinear optomechanical resonance entering a self-organized energy transfer pattern. *Chaos Soliton. Fract.* **173**, 113624 (2023).

44. X. Gu, J. Zhang, S. Ding, X. Jiang, B. He, Q. Lin, Optical frequency comb significantly spanned to broadband by an optomechanical resonance. *Photonics Res.* **12**, 1981–1990 (2024).

45. Y. Wang, M. Zhang, Z. Shen, G.-T. Xu, R. Niu, F.-W. Sun, G.-C. Guo, C.-H. Dong, Optomechanical frequency comb based on multiple nonlinear dynamics. *Phys. Rev. Lett.* **132**, 163603 (2024).

46. J.-C. Shi, Q.-X. Ji, Q.-T. Cao, Y. Yu, W. Liu, Q. Gong, Y.-F. Xiao, Vibrational Kerr solitons in an optomechanical microresonator. *Phys. Rev. Lett.* **128**, 073901 (2022).

47. K. Jia, X. Wang, D. Kwon, J. Wang, E. Tsao, H. Liu, X. Ni, J. Guo, M. Yang, X. Jiang, J. Kim, S.-N. Zhu, Z. Xie, S.-W. Huang, Photonic flywheel in a monolithic fiber resonator. *Phys. Rev. Lett.* **125**, 143902 (2020).

48. Y. Bai, M. Zhang, Q. Shi, S. Ding, Y. Qin, Z. Xie, X. Jiang, M. Xiao, Brillouin-Kerr soliton frequency combs in an optical microresonator. *Phys. Rev. Lett.* **126**, 063901 (2021).

49. J. K. Jang, A. Klenner, X. Ji, Y. Okawachi, M. Lipson, A. L. Gaeta, Synchronization of coupled optical microresonators. *Nat. Photonics* **12**, 688–693 (2018).

50. J. K. Jang, X. Ji, C. Joshi, Y. Okawachi, M. Lipson, A. L. Gaeta, Observation of Arnold tongues in coupled soliton Kerr frequency combs. *Phys. Rev. Lett.* **123**, 153901 (2019).

51. J. Li, S. Wan, Y. Wang, R. Niu, M. Li, Z. Shen, G.-C. Guo, C.-H. Dong, Synchronization of optomechanical oscillator and breathing soliton. *Laser Photonics Rev.* **17**, 2200496 (2023).

52. D. K. Armani, T. J. Kippenberg, S. M. Spillane, K. J. Vahala, Ultra-high-*Q* toroid microcavity on a chip. *Nature* **421**, 925–928 (2003).

53. J. Ma, X. Jiang, M. Xiao, Kerr frequency combs in large-size, ultra-high-*Q* toroid microcavities with low repetition rates. *Photonics Res.* **5**, B54–B58 (2017).

54. T. J. Kippenberg, S. M. Spillane, K. J. Vahala, Kerr-nonlinearity optical parametric oscillation in an ultrahigh-Q toroid microcavity. *Phys. Rev. Lett.* **93**, 083904 (2004).

55. M. Tomes, T. Carmon, Photonic micro-electromechanical systems vibrating at X-band (11-GHz) rates. *Phys. Rev. Lett.* **102**, 113601 (2009).

56. C. Bao, J. A. Jaramillo-Villegas, Y. Xuan, D. E. Leaird, M. Qi, A. M. Weiner, Observation of Fermi-Pasta-Ulam recurrence induced by breather solitons in an optical microresonator. *Phys. Rev. Lett.* **117**, 163901 (2016).

57. E. Lucas, M. Karpov, H. Guo, M. L. Gorodetsky, T. J. Kippenberg, Breathing dissipative solitons in optical microresonators. *Nat. Commun.* **8**, 736 (2017).

58. M. Yu, J. K. Jang, Y. Okawachi, A. G. Griffith, K. Luke, S. A. Miller, X. Ji, M. Lipson, A. L. Gaeta, Breather soliton dynamics in microresonators. *Nat. Commun.* **8**, 14569 (2017).

59. J. Rosenberg, Q. Lin, O. Painter, Static and dynamic wavelength routing via the gradient optical force. *Nat. Photonics* **3**, 478–483 (2009).

60. S. Boccaletti, A. N. Pisarchik, C. I. Del Genio, A. Amann, *Synchronization: From Coupled Systems to Complex Networks* (Cambridge Univ. Press, Cambridge, 2018).

61. M. Zhang, G. S. Wiederhecker, S. Manipatruni, A. Barnard, P. McEuen, M. Lipson, Synchronization of micromechanical oscillators using light. *Phys. Rev. Lett.* **109**, 233906 (2012).

62. M. Bagheri, M. Poot, L. Fan, F. Marquardt, H. X. Tang, Photonic cavity synchronization of nanomechanical oscillators. *Phys. Rev. Lett.* **111**, 213902 (2013).

63. J. Sheng, X. Wei, C. Yang, H. Wu, Self-organized synchronization of phonon lasers. *Phys. Rev. Lett.* **124**, 053604 (2020).

64. P. Liao, K. Zou, C. Bao, A. Kordts, M. Karpov, M. H. P. Pfeiffer, L. Zhang, Y. Cao, A. Almaiman, F. Alishashi, A. Mohajerin-Ariaei, A. Fallahpour, M. Tur, T. J. Kippenberg, A. E. Willner, Chip-scale dual-comb source using a breathing soliton with an increased resolution. in *Conference on Lasers and Electro-Optics.* paper JTh5A.4 (Optical Society of America, 2018).

65. D. C. Cole, S. B. Papp, Subharmonic entrainment of kerr breather solitons. *Phys. Rev. Lett.* **123**, 173904 (2019).

66. M. R. Vanner, I. Pikovski, G. D. Cole, M. S. Kim, Č. Brukner, K. Hammerer, G. J. Milburn, M. Aspelmeyer, Pulsed quantum optomechanics. *Proc. Natl. Acad. Sci. U.S.A.* **108**, 16182–16187 (2011).

67. J. Zhang, B. Peng, S. Kim, F. Monifi, X. Jiang, Y. Li, P. Yu, L. Liu, Y.-X. Liu, A. Alù, L. Yang, Optomechanical dissipative solitons. *Nature* **600**, 75–80 (2021).

68. T. Hansson, D. Modotto, S. Wabnitz, On the numerical simulation of Kerr frequency combs using coupled mode equations. *Opt. Commun.* **312**, 134–136 (2014).
